# Isatuximab, carfilzomib, lenalidomide, and dexamethasone (Isa-KRd) in front-line treatment of high-risk multiple myeloma: interim analysis of the GMMG-CONCEPT trial

**DOI:** 10.1038/s41375-021-01431-x

**Published:** 2021-11-03

**Authors:** Lisa B. Leypoldt, Britta Besemer, Anne Marie Asemissen, Mathias Hänel, Igor Wolfgang Blau, Martin Görner, Yon-Dschun Ko, Hans Christian Reinhardt, Peter Staib, Christoph Mann, Raphael Lutz, Markus Munder, Ullrich Graeven, Rudolf Peceny, Hans Salwender, Anna Jauch, Manola Zago, Axel Benner, Diana Tichy, Carsten Bokemeyer, Hartmut Goldschmidt, Katja C. Weisel

**Affiliations:** 1 Department of Hematology, Oncology and Bone Marrow Transplantation with Section of Pneumology, University Medical Center Hamburg-Eppendorf, Hamburg, Germany; 2 Department of Hematology, Oncology, Immunology, Rheumatology and Pulmonology, University Hospital of Tuebingen, Tuebingen, Germany; 3 Department of Hematology, Oncology and Bone Marrow Transplantation, Klinikum Chemnitz, Chemnitz, Germany; 4 Department of Internal Medicine, Charité University Medicine Berlin, Berlin, Germany; 5 Department of Hematology, Oncology and Palliative Care, Klinikum Bielefeld Mitte, Bielefeld, Germany; 6 Department of Internal Medicine, Hematology and Oncology, Johanniter Krankenhaus Bonn, Bonn, Germany; 7 Department of Hematology and Stem Cell Transplantation, University Hospital Essen, University Duisburg-Essen, German Cancer Consortium (DKTK partner site Essen), Essen, Germany; 8 Department of Hematology and Oncology, St. Antonius Hospital Eschweiler, Eschweiler, Germany; 9 Department of Hematology, Oncology and Immunology, University Hospital of Gießen and Marburg, Marburg, Germany; 10 University Hospital Heidelberg, Internal Medicine V and National Center for Tumor Diseases (NCT), Heidelberg, Germany; 11 Department of Internal Medicine III, University Medical Center Mainz, Mainz, Germany; 12 Department of Hematology, Oncology and Gastroenterology, Maria Hilf Kliniken, Mönchengladbach, Germany; 13 Department of Oncology, Hematology and Stem Cell Transplantation, Klinikum Osnabrück, Osnabrück, Germany; 14 Asklepios Tumorzentrum Hamburg, AK Altona and AK St. Georg, Hamburg, Germany; 15 Institute of Human Genetics, University of Heidelberg, Heidelberg, Germany; 16 Center for Clinical Trials, University Hospital of Tuebingen, Tuebingen, Germany; 17 Division of Biostatistics, German Cancer Research Center (DKFZ) Heidelberg, Heidelberg, Germany

**Keywords:** Phase II trials, Myeloma

The continuous implementation of novel agents in the treatment of multiple myeloma (MM) has led to significant improvement in survival. Especially the addition of monoclonal antibodies directed against CD38 to standard of care regimens led to significantly deepening responses and improved survival outcomes [1]. However, treatment of high-risk (HR) MM remains challenging with still markedly impaired survival, and risk-adapted treatment concepts are rare [2, 3]. Even aggressive approaches resulted in two-year median progression-free survival (PFS) rates of approximately 50% [4]. The GMMG-CONCEPT trial (NCT03104842) investigates the quadruplet regimen isatuximab, carfilzomib, lenalidomide, and dexamethasone (Isa-KRd) in front-line treatment of solely HRMM. Here, we report the interim analysis (IA) focusing on best response during induction and presenting first data on PFS of the first 50 patients.

The IA reports on the first 50 patients included in this phase II, open-label, two-arm, multi-center clinical trial with planned recruitment of 246 patients. Patients were eligible if they had ND symptomatic MM according to international consensus criteria with HR features, defined by the presence of del17p (≥10% of purified cells) or t(4;14) or t(14;16) or > 3 copies of 1q21. Furthermore, all patients had to have ISS II or III stage disease [5]. Prior MM-specific treatment was allowed as emergency treatment with a maximum of one cycle of any anti-MM first-line treatment. All patients received ECG and ECHO at screening.

Patients were openly assigned to study arms according to age and transplant eligibility (arm A: patients ≤ 70 years and eligible for HDT; arm B: patients > 70 years). Study treatment consisted of six cycles Isa-KRd induction, four cycles Isa-KRd consolidation, and Isa-KR maintenance. Transplant-eligible patients underwent HDT with autologous stem cell transplantation (ASCT) after stem cell collection, transplant-ineligible patients received two additional Isa-KRd induction cycles. Primary endpoint of this trial is achievement of minimal residual disease (MRD) negativity measured by next-generation flow after consolidation. Induction treatment with Isa-KRd consisted of six 28-day-cycles with isatuximab 10 mg/kg of body weight intravenously (i.v.) weekly during the first and on day 1 and 15 of any subsequent cycle, carfilzomib 20 mg/m^2^ of body surface area i.v. on day 1 and 2 of the first and 36 mg/m^2^ i.v. on day 8, 9, 15, 16 of the first and day 1, 2, 8, 9, 15, 16 of any subsequent cycle, lenalidomide 25 mg orally (p.o.) on day 1–21 of all cycles, and dexamethasone 40 mg (20 mg for subjects >75 years of age) p.o./i.v. on day 1,8,15,22 of all cycles. Prophylactic anticoagulation was obligate and chosen upon the investigator’s decision. The population for this IA on overall response rate (ORR) at the end of induction includes the first 50 enrolled patients who received at least one cycle of induction treatment and were eligible for at least one response assessment (46 patients in arm A and 4 patients in arm B). Overall response was determined as the best response until the end of induction including mobilization. Median age was 58 (range: 42–82) years. 56% of patients showed ISS stage II, 44% ISS stage III disease. The most common cytogenetic aberration defining HR disease was del17p in 52% of patients followed by >3 copies of 1q21 in 42%, t(4;14) in 38% and t(14;16) in 12%, respectively. 15 patients (30%) showed ≥2 HR aberrations and 20% of patients had an elevated LDH.

Forty-four of 50 patients completed induction, seven patients discontinued treatment due to progressive disease (*n* = 3), death (*n* = 3) or patient’s request (*n* = 1). Average dose intensities were 95.7% for isatuximab, 95.2% for dexamethasone, 91.6% for carfilzomib, and 87.9% for lenalidomide. With regards to the goal of this IA reporting on best response during induction, all patients (50/50; ORR = 100%) responded to the induction treatment showing at least a partial response (PR) as best response. 45/50 patients (90%) showed a VGPR or better, 20/50 patients (40%) a complete response (CR) and three patients (6%) a stringent complete response (sCR) (Table 1). Of the four patients in treatment arm B, all patients completed induction and achieved VGPR (Table 1).

**Table 1:** Best response during induction.

	Arm A, *N* = 46 (%)	Arm B, *N* = 4 (%)	Overall, *N* = 50 (%)
≥CR	23 (50)	0	23 (46)
sCR	3 (6.5)	0	3 (6)
CR	20 (43.5)	0	20 (40)
VGPR	18 (39.1)	4 (100)	22 (44)
PR	5 (10.9)	0	5 (10)
ORR	46 (100)	4 (100)	50 (100)
≥VGPR	41 (89.1)	4 (100)	45 (90)
MRD negative	20/31 (64.5)	0/1 (0)	20/32 (62.5)

**Fig. 1:**
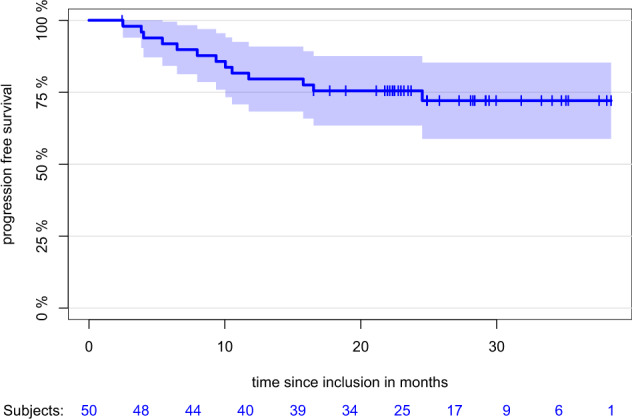
Progression-free survival (PFS) of the IA population (*N* = 50). With a median follow-up of 24.9 months, median PFS was not reached with a median 12-month PFS of 79.6% (CI: 68.3%; 90.9%) and a median 24-month PFS of 75.5% (CI: 63.5%; 87.6%).

Median time to first response was 34 days with 95.8% achieving ≥PR after the first induction cycle. Assessment of MRD during induction was recommended in all patients achieving ≥VGPR. In total, 33 patients underwent MRD assessment. Of those, 20 patients were negative, 11 patients positive, two patients were non-assessable. After a median follow-up of 24.9 months, median PFS was not reached with a median 12-month PFS of 79.6% (CI: 68.3%; 90.9%) and a median 24-month PFS of 75.5% (CI: 63.5%; 87.6%) (Fig. 1). Most common adverse events (AE) of any grade occurring in ≥ 10% of patients were neutropenia, lymphopenia, leukopenia, anemia, thrombocytopenia, upper respiratory tract infections, pyrexia, rash, peripheral sensory neuropathy, arterial hypertension, and nasopharyngitis. Most common AEs grade 3/4 occurring in ≥10% of patients were neutropenia, lymphopenia, leukopenia, thrombocytopenia, anemia, infections, and arterial hypertension. Serious adverse events (SAE) of ≥grade 3 occurred in 18 patients, most common SAEs being infectious (*n* = 5) and cardiovascular disorders (*n* = 5). Grade 3/4 cardiac failure was documented in 4 patients, isatuximab-related infusion reactions occured in 32%, all grade 1 or 2. Death on study during induction phase including mobilization occurred in three patients with two fatal pneumonias and one fatal neutropenic sepsis after stem cell mobilization. Median number of collected CD34 + cells was 6.0 × 10^6^ per kg body weight.

Trials for solely HRMM are rare and the proportion of HR patients in first-line phase III trials is generally limited representing around 15–25% of the total patient population [6, 7]. Even more, a substantial proportion of ultra HR patients does not enter clinical trials due to aggressiveness of the disease leading to emergency treatment before potential trial inclusion. With one cycle of any myeloma-directed therapy being allowed before enrollment, the GMMG-CONCEPT trial enabled even ultra HR patients including plasma cell leukemia and patients primary non-responding to a first treatment cycle to be included. GMMG-CONCEPT is the first trial investigating the Isa-KRd quadruplet regimen in the treatment of MM. This IA focusing on best overall response during induction showed an ORR of 100% with 90% of patients achieving ≥ VGPR and 46% showing a CR or sCR and so revealed promising results with no patients primary refractory to the chosen quadruplet combination. MRD analysis during induction was not obligate, however, recommended for all patients achieving at least a VGPR. Of 33 patients tested for MRD at this early time point, 31 were evaluable and of those, 20 were negative for MRD. To address the question whether the achieved early high response rates translate into survival outcome, we conducted a PFS analysis after a median follow-up of 24.9 months demonstrating a two-year PFS rate of 75.5% with a median PFS not reached. To the best of our knowledge, this is one of the highest described in this unfavorable patient group. Isa-KRd as a quadruplet regimen was tolerable, AEs were clinically manageable and consistent with known toxicities of each individual substance. Reported AEs of interest, especially cardiac toxicities, were within the expected range, rates of peripheral neuropathy were low. Regarding the 3 reported fatal infectious events, with one where a relation to study medication could not be excluded, a careful look on the larger patient population is needed. However, in this difficult-to-treat population, we see a positive risk-benefit analysis outweighing efficacy above toxicity. Currently, there are several trials underway investigating quadruplet regimens in NDMM and even HRMM. In the SWOG 1211 trial for HR patients, addition of the monoclonal anti-SLAMF7 antibody elotuzumab to bortezomib, lenalidomide and dexamethasone did not lead to improved outcome [8]. ORR was 83% with a 2.1% CR and a 21.3% VGPR rate in the quadruplet treatment arm, median PFS was 31.47 months [8]. Out of the FORTE trial, Gay and colleagues reported a PFS rate of 62% after four years in HR patients treated with upfront KRd, ASCT, and KR or R maintenance, however using a broad definition of HR accounting for more than 50% of the trial population [9]. The UK OPTIMUM HR study investigating quintruplet induction of Dara-CVRD followed by HDT and ASCT in HR patients most recently reported an ORR of 94% with a ≥ VGPR rate of 80% as the best response during induction [10]. Two recent trials are investigating the anti-CD38-KRd (Dara-KRd) combination not restricted to HR patients: The single-center MANHATTAN trial reported an ORR of 100% with a 1-year-PFS of 98% in 41 patients [11]. The MASTER trial showed a rate of 90% ≥VGPR after induction in 70 patients [12]. Taken together, anti-CD38-KRd trials particularly underline the high potential in achieving deep responses including MRD-negativity. This might open again the discussion about the future relevance of primary HDT and ASCT.

In summary, our data demonstrate encouraging rates of rapid and deep remissions in HR MM patients with Isa-KRd induction, which may translate into durable responses in this difficult-to-treat patient group and is supported by the first survival data on PFS with a two-year PFS rate of 75.5%. The trial completed recruitment of the first population of 153 patients early in 2020 and is ongoing with an expansion cohort for a total of 246 patients. Further results will be reported.
